# A parameterized gamma-variate function can describe the temporal response of cerebral blood flow after Acetazolamide injection in Moyamoya patients

**DOI:** 10.1016/j.ynirp.2026.100359

**Published:** 2026-05-21

**Authors:** Joao M. Sousa, Teodor Svedung Wettervik, Fartein Velle, Alex A. Bhogal, Johan Berglund, Per Enblad, Johan Wikström, Anders Lewén, Markus Fahlström

**Affiliations:** a Department of Medical Physics, Uppsala University Hospital, Uppsala, Sweden; b Department of Surgical Sciences, Molecular Imaging and Medical Physics, Uppsala University, Uppsala, Sweden; c Department of Medical Sciences, Neurosurgery, Uppsala University, Uppsala, Sweden; d Translational Neuroimaging Group, Center for Imaging Science, University Medical Center Utrecht, Utrecht University, the Netherlands; e Department of Surgical Sciences, Neuroradiology, Uppsala University, Uppsala, Sweden

**Keywords:** Acetazolamide, Arterial spin labeling, Cerebral blood flow, Cerebrovascular reserve, Modelling, Moyamoya

## Abstract

**Background:**

Quantitative modelling of the temporal response of cerebral blood flow (CBF) following Acetazolamide (ACZ) may be performed using a parameterized gamma-variate function with physiologically interpretable parameters. When combined with sequential CBF measurement before and after administration of ACZ, such a model could enable assessment of cerebrovascular reserve (CVR) while accounting for regional differences in the timing of the vascular response between steno-occlusive and unaffected territories. To evaluate this approach, we applied the model to patients with moyamoya disease (MMD).

**Methods:**

Twenty-four MMD patients underwent MRI including structural- and sequential multi-delay arterial spin labeling acquisitions before and after ACZ injection. Non-linear least squares curve fitting was performed on CBF values extracted from vascular regions. Root mean squared error (RMSE), absolute and relative CVR (CVR_diff_ and CVR_rel_) and time to maximum CBF response (t_max_) were compared between the anterior circulation (affected) and posterior circulation (unaffected).

**Results:**

Twenty-one patients were evaluated (age range 10-61; 16 females), three was excluded due to technical issues. Median RMSE ranged between 2.8 and 3.8 ml/100 g/min. CVR_rel_ was lower in anterior regions compared to posterior regions (mean differences 10.4-29.2%), whereas, CVR_diff_ differences were in general similar (0.8-6.9 ml/100 g/min). Median difference in t_max_ of 2.3-6.8 min was observed in affected compared to unaffected regions. Reversible intracerebral steal was found in two patients and successfully modelled using an extended gamma-variate function.

**Conclusion:**

This parameterized gamma-variate function framework provides physiologically interpretable parameters for modelling the CBF response to ACZ in MMD.

## Highlights

• A parameterized gamma-variate models the temporal CBF response to Acetazolamide.
• The framework is extended to model reversible intracerebral steal.
• Peak CBF response is delayed in affected vs. unaffected regions in MMD.
• Single-time-point CVR underestimates model-based CVR in all regions.

## 1 Introduction

The cerebrovascular reserve (CVR) is defined as the brain's capacity to increase cerebral blood flow (CBF) by vasodilation in response to a vasoactive stimulus (Powers, 1991). Chronic vasodilation may be present in patients with steno-occlusive conditions such as moyamoya disease (MMD) or syndrome (MMS) to compensate for the decrease in CBF secondary to impaired cerebral perfusion pressure distal to the occlusion (Scott and Smith, 2009). Consequently, the remaining CVR capacity is reduced, which limits the ability to further regulate CBF and is associated with an increased risk of stroke and transient ischemic attacks (Gupta et al., 2012; Markus and Cullinane, 2001). An additional risk is seen in patients with intracerebral steal phenomenon where vasodilatory provocation leads to a paradoxical reduction in CBF, indicating a negative CVR (Poublanc et al., 2013; Vorstrup et al., 1986; Webster et al., 1995; Yonas et al., 1993). Furthermore, studies have also reported the so-called “reversible” intracerebral steal – a dip-then-recovery pattern where an early reduction in CBF is followed by an increase compared to baseline (Fahlstrom et al., 2024; Kobayashi et al., 2021; Kuwabara et al., 1995; Poublanc et al., 2013; van Niftrik et al., 2023). The later phenomenon is rarely reported in the literature and therefore remains poorly understood; its clinical implications have not been evaluated.

CVR can be estimated using acetazolamide (ACZ), a carbonic anhydrase inhibitor that produces a long-lasting vasodilatory response (Okazawa et al., 2001) where CVR is calculated as the highest relative- or absolute change in CBF after injection of ACZ (Dahl et al., 1992; Vagal et al., 2009). Inherently a temporal response, CBF does not remain constant during the provocation, hence the estimated CVR will vary depending on when the post-injection measurement is performed (Dogra et al., 2023; Fahlstrom et al., 2024; Hartkamp et al., 2012). Furthermore, studies have demonstrated that the temporal response of CBF to ACZ differs between healthy controls and patients with cerebrovascular diseases, as well as between affected and unaffected vascular regions within patients (Dogra et al., 2023; Fahlstrom et al., 2024; Ficzere et al., 1997; Hartkamp et al., 2012; Stoll et al., 1996), suggesting that a dynamic approach to measure CBF following ACZ injection is preferred over a static CVR measurement. A previous study proposed a bi-exponential model to describe the temporal response in CBF after ACZ injection based on multiple consecutive measurements using single-delay Arterial Spin Labeling (ASL) (Fahlstrom et al., 2024). The bi-exponential model adapted well to a dataset of patients with MMD. However, its application was limited by the lack of distinct physiological interpretation and constraint to an upper limit of CVR estimates justifying further elaboration. Together with other methodological concerns such as inherent limitations using single-delay ASL-based acquisition in patients with cerebrovascular disease and limited number of datapoints used for model fitting warranted for further development and improvements on the existing methodology.

In this study we present an alternative parametrization of the gamma-variate function to describe the temporal response of CVR following ACZ injection. We provide an assessment of this gamma-variate function in patients with MMD. Lastly, we extend the model to describe reversible intracerebral steal in two cases to increase our understanding of this phenomenon.

## 2 Methods

### 2.1 Model description

The expression for the gamma-variate function is

(1) $$\begin{array}{c} C\left(A,\alpha ,\beta ,t\right)=At^{\alpha }\;\exp\left(-\frac{t}{\beta }\right)\text{,} \end{array}$$

where A is amplitude, *α* is shape, *β* is rate and t is time (min). Using the derivation in Madsen (1992) we can parameterize equation (1) as

(2) $$\begin{array}{c} G\left(y_{\max},t_{\max},\alpha ,t\right)=y_{\max}\;{\left(\frac{t}{t_{\max}}\exp\left(1-\frac{t}{t_{\max}}\right)\right)}^{\alpha }\text{,} \end{array}$$

where y_max_ is the maximum value of the function, which occurs at time t_max_ (min). We use this to describe the CBF response following ACZ injection (CBF_ACZ_ in ml/100 g/min) in relation to CBF before injection (CBF_pre_ in ml/100 g/min), substituting y_max_ with CVR_diff_:

(3) $$\begin{array}{c} {CBF}_{ACZ}\left(t\right)={CBF}_{pre}+{CVR}_{diff}{\left(\frac{t}{t_{\max}}\exp\left(1-\frac{t}{t_{\max}}\right)\right)}^{\alpha } \end{array}$$

where CVR_diff_ is the peak increase in CBF after ACZ injection (ml/100 g/min) and t_max_ is the time from injection to the peak (min) (see Supplemental Fig. S1A). With this parametrization, the fitted parameters directly correspond to physiologically meaningful features of the temporal CBF response to ACZ. Relative CVR in percent (CVR_rel_) can be calculated as ${\text{CVR}}_{\text{diff}}/{\text{CBF}}_{\text{pre}}\cdot 100$. In this study, we use CVR_diff_ for the absolute CBF increase following ACZ (ml/100 g/min) and CVR_rel_ for the same increase normalized to baseline CBF (%). This follows the convention commonly used in the positron emission tomography (PET) and ASL literature (Juttukonda and Donahue, 2019; Okazawa et al., 2001), and differs from CVR estimated by Blood Oxygen Level Dependent (BOLD) imaging, where CVR is typically expressed per unit change in end-tidal CO_2_ (%/mmHg) (Sleight et al., 2021). Both measures are reported because CVR_rel_ may be dependent on baseline CBF (Afzali-Hashemi et al., 2021; Dahl et al., 1994).

#### 2.1.1 Modelling of reversible intracerebral steal

Intracerebral steal is conventionally defined as a decrease in CBF after ACZ injection that remains below CBF_pre_ throughout the vasodilatory challenge (Vorstrup et al., 1986). Reversible intracerebral steal, in contrast, presents as an initial decrease in CBF after ACZ injection followed by a recovery above CBF_pre_ – the dip-then-recovery pattern (Kobayashi et al., 2021; Kuwabara et al., 1995; Poublanc et al., 2013); examples are provided in Fig. 2, Fig. 3. We model this behavior using a two-component coupled gamma-variate model adapted from the formulation of Davenport (1983) for organ blood flow and recirculation. The recovery component takes the form of equation (1) – the original gamma-variate response on which the framework is based – while the opposing steal component subtracts from it. Adding CBF_pre_ as the baseline offset gives:

(4) $$\begin{array}{c} {CBF}_{steal}\left(t\right)={CBF}_{pre}-Bt^{\left(\alpha -1\right)/2}\;\exp\left(-\frac{t}{\beta }\right)+At^{\alpha }\;\exp\left(-\frac{t}{\beta }\right) \end{array}$$

The two components share shape (*α*) and rate (*β*) parameters, reflecting that they represent coupled manifestations of the same response rather than independent processes. The steal component has shape (α−1)/2 and dominates early, producing the initial decline in CBF below CBF_pre_ that is characteristic of reversible intracerebral steal; the recovery component has shape *α* and drives CBF above CBF_pre_ at later times. We adopt the functional form of Davenport's two-component gamma-variate as a flexible description of the dip-then-recovery pattern, rather than as a literal causal model of the steal process. The time delay T present in the original formulation is set to zero since both components arise from the same triggering event (ACZ injection).

Because the two components interact, the fitted parameters A, B, *α* and *β* do not correspond directly to features of the composite response as they did in equation (3). In the case of reversible intracerebral steal, we are interested in four features: CVR_diff_, t_max_, CVR_steal_ (largest absolute decrease in CBF in ml/100 g/min) and t_min_ (time from injection to CVR_steal_ in min; see Supplemental Fig. S1B). These are derived from the fitted function by numerical approximation.

### 2.2 Patients

All consecutive MMD or MMS patients examined with multi-delay (MD)-ASL before and after ACZ injection at Uppsala University Hospital between May 2025 and March 2026 (n = 24). Patients with previous revascularization surgery and non-operated patients were both eligible. The Suzuki score system was used to grade the patient's stage of disease by an experienced neuroradiologist based on a magnetic resonance angiography (MRA) acquired as a part of the complete MR protocol. All patients were intravenously injected with ACZ (1000 mg for adults and 10 mg/kg for children). The study was performed in accordance with the Declaration of Helsinki and was approved by the Swedish Ethical Review Authority (Ref. no. 2019-01316, date of approval 2019-07-13). All included patients or legal guardians signed an informed consent form. This study was retrospective and not pre-registered.

### 2.3 Magnetic resonance imaging

All examinations were performed on a 3.0 T MRI system (dStream Achieva, Philips Healthcare, Best, the Netherlands) using a 32-channel head coil. Image acquisitions were identical for all patients throughout the study period. CBF measurements were acquired using a MD-ASL sequence with pseudo-continuous labeling, variable repetition time, optimized background suppression and segmented 3D gradient and spin-echo readout (GRASE) (Mikayama et al., 2025; Togao et al., 2023). Improved motion-sensitized driven-equilibrium was applied for arterial signal suppression (Obara et al., 2014). Acquisition was performed with six combinations of label- and post-label durations. Full set of acquisition details can be found in Supplemental Table S1. A calibration image was acquired with repetition time (TR) 3000 ms and otherwise identical acquisition parameters. The labelling plane was placed perpendicular to the internal carotid arteries based on a phase-contrast MRA survey. Scan time for the MD-ASL and calibration image was 5 min 15 s and 55 s, respectively. A total of five MD-ASL datasets including calibration images were acquired during each examination; one prior to ACZ injection and four starting at 2-, 9-, 16- and 25-min post-injection. The examination also included a 3D T1-weighted (T1w) image for anatomical processing and registration and a time-of-flight MRA for Suzuki grading as described above.

### 2.4 Data processing

#### 2.4.1 Structural image processing

Cortical reconstructions and volumetric segmentations were performed with the Freesurfer image analysis suite version 7.4.1 (http://surfer.nmr.mgh.harvard.edu/) using a T1w image for each patient.

#### 2.4.2 ASL image processing

QASL (version 1.2.0, Quantified Imaging, London, UK) was used for MD-ASL image processing, including motion correction, perfusion quantification, calibration and registration to structural and standard spaces via ANTs version 0.5.4. Perfusion quantification including macrovascular correction was performed using Structured Stochastic Variational Bayes (SSVB) (Kirk et al., 2025). The calibration image was corrected for short TR (Pinto et al., 2020) and a blood-tissue partition coefficient of λ = 0.9, label efficiency of α_label_ = 0.85 and background suppression efficiency of α_bgs_ = 0.80 (this were applied to obtain CBF measures in absolute units of ml/100 g/min).

#### 2.4.3 Data analysis

A standard space vascular atlas defining four supra- and infratentorial arterial brain regions in standard space (Liu et al., 2023): anterior- (ACA), middle- (MCA), posterior cerebral artery (PCA) and vertebrobasilar artery (VBA) was transformed from standard to each subject's structural space using nearest neighbor interpolation by inversion of the registration matrix produced by the QASL pipeline. A grey matter mask (GM) was defined based on the Freesurfer volumetric segmentations. The GM mask was multiplied with the structural vascular atlas to remove any contributions from white matter and cerebrospinal fluid. A representative T1w image with masked vascular regions is presented in Supplemental Fig. S2. Regional mean CBF values were consecutively extracted for each time point. Furthermore, disease grading, based on the Suzuki score system as described above, were assigned to each hemisphere. CVR maps, for visualization were calculated by dividing post-ACZ injection CBF maps to the pre-ACZ injection CBF map whereafter a smoothing filter was applied.

### 2.5 Curve fitting

Curve fitting was performed, based on the extracted regional mean CBF values, using least squares regression as implemented in GraphPad Prism 10 for Mac (GraphPad Software, La Jolla, CA, USA). First, equation (3) was used with initial values (constraints) set to *CBF*_*pre*_ = 80 ml/100 g/min (0 to 140 ml/100 g/min), *CVR*_*diff*_ = 40 ml/100 g/min (<80 ml/100 g/min), *t*_max_ = 20 min (no constraint) and *α* = 1 (>0.5).

All curve fits were inspected visually to identify vascular regions with reversible intracerebral steal defined by two criteria: at least one post-injection CBF value below CBF_pre_, followed by subsequent values above CBF_pre_. Ambiguous fits were identified by outlier analysis on the best-fit t_max_ values across all patients and vascular regions without reversible intracerebral steal using robust regression and outlier removal (ROUT, false discovery rate 1%) as implemented in Graphpad Prism 10. Curve fits flagged as t_max_ outliers consistently lacked a local maximum in the sequential mean CBF values, reflecting insufficient sampling to constrain t_max_. Parameters values from ambiguous fits were not included in the statistical analysis and were evaluated separately.

Vascular regions with reversible intracerebral steal were re-fitted using equation (4) with CBF_pre_ as measured CBF before ACZ injection and initial values set to A = 10 and B = 1, both in arbitrary units and unconstrained, *β* = 4 min (no constraint) and *α* = 2 (>1). CVR_diff_, t_max_, t_min_ and CVR_steal_ were calculated by numeric approximation.

Time points used for all curve fittings were 0, 5, 12, 19 and 28 min corresponding to the middle for each ASL data acquisition, including calibration image, in relation to the ACZ injection.

### 2.6 Statistics

Left and right vascular regions were pooled together. Patients with suspected reversible intracerebral steal and ambiguous curve fits were excluded from the statistical analysis and are instead considered on a case-by-case basis. Furthermore, unaffected ACA/MCA regions in patients with unilateral disease were excluded. The D'Agostino-Pearson omnibus test was performed to test for normality. Descriptive statistics are expressed as mean (standard deviation, SD) for normal distributed data or median (interquartile range, IQR) for non-normal distributed data. Derived p-values are two-sided and presented as exact values or p < 0.001, p < 0.05 being considered significant. GraphPad Prism 10 for Mac was used for statistical analysis and graph design.

#### 2.6.1 Curve fitting performance

Curve fitting performance was assessed by calculating the root mean squared error (RMSE). RMSE passed the test for normality.

#### 2.6.2 Analysis of parameters

CBF_pre_ and CVR_rel_ passed the test for normality whereas CVR_diff_ and t_max_ did not. To test whether any differences were present between vascular regions a repeated measure ANOVA test with Sidak's correction was performed on CVR_rel_ and CBF_pre_ and a Friedman's test with Dunn's test was performed on CVR_diff_ and t_max_. Post-hoc analysis was conducted on pairs ACA vs PCA, MCA vs PCA, ACA vs VBA and MCA vs VBA, corresponding to possible combinations of affected-unaffected pairs. Both repeated measure ANOVA and Friedman's test does not handle missing values, hence, patients with missing regional values were excluded from the post-hoc analysis. Model-based CVR (CVR_rel_ and CVR_diff_) and static CVR were compared per vascular region. Static CVR was derived from CBF acquisition starting at 16 min post-ACZ injection (CVR_rel, 16min_ and CVR_diff, 16min_). Consistent with the primary analysis, CVR_rel_ was tested with the paired *t*-test and CVR_diff_ with the Wilcoxson signed-rank test. Correction for multiple comparisons was not applied, as each test addresses a pre-specific directional hypothesis that the static measurement underestimates the model-based value.

## 3 Results

### 3.1 Patient characteristics

Twenty-one patients with MMD or MMS (average age at examination, 41 years; range 10 to 61; 5 males, 16 females) were included after exclusions. Three patients were excluded; two due to severe motion artefacts and one due to ineffective labelling. Seventeen patients were diagnosed with bilateral disease and four patients were diagnosed with unilateral disease.

Indirect revascularization surgery had been performed in five patients and direct revascularization in two patients at the time of each patient's examination. Four ambiguous fits were identified in four patients. Seven vascular regions in two patients presented with reversible intracerebral steal. Patient characteristics and flowchart describing patient inclusion/exclusion is presented in Supplemental Table S2 and Fig. S3, respectively.

### 3.2 Analysis of parameters

**Figure 1:**
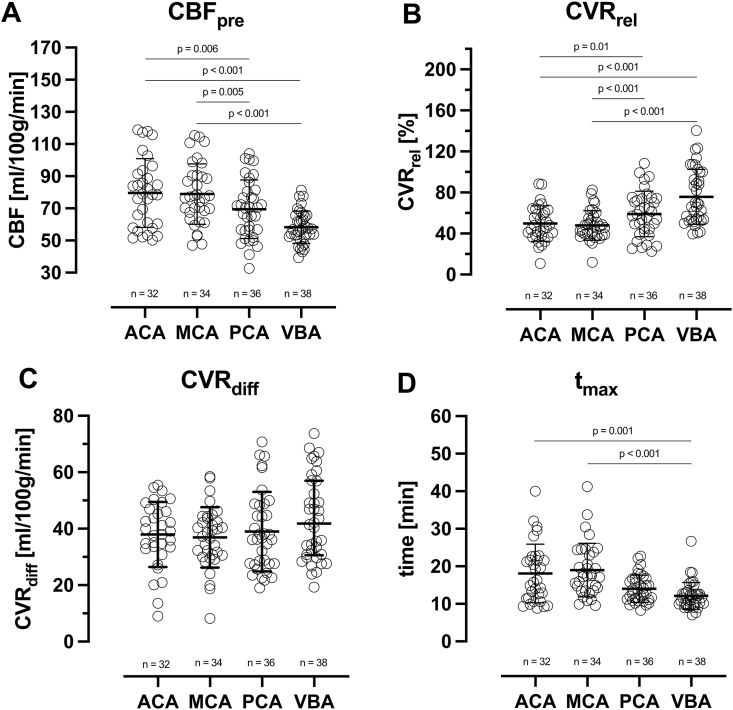
Scatter-plots presenting CBF_pre_ (A), CVR_rel_ (B), CVR_diff_ (C) and t_max_ (D) for all vascular regions, respectively. Asterix (∗) indicates if difference between affected (ACA, MCA) and unaffected (PCA, VBA) regions are significant. Bars represent mean and standard deviation for CVR and median and interquartile range for t_max_. Left and right vascular regions are pooled together, unaffected ACA/MCA and affected PCA regions were excluded, number of vascular regions included in each descriptive analysis is indicated by n. Abbreviations: ACA, anterior cerebral artery; CBF, Cerebral blood flow; CVR, cerebrovascular reserve; MCA, middle cerebral artery; PCA, posterior cerebral artery; VBA, vertebra-basilar artery.

Table 1, Table 2 and Fig. 1 presents descriptive statistics of all analyzed parameters and results from statistical tests. Median RMSE values across all vascular regions were generally low with an average error of around 4% to 5% relative to mean CBF_pre_ (see Table 1). Median RMSE varied between 2.8 and 3.8 ml/100 g/min for all vascular regions together indicating a generally good curve fitting performance. CBF_pre_ in ml/100 g/min, was significantly higher in ACA and MCA (mean ± SD; 79.5 ± 21.4 and 78.9 ± 18.8 ml/100 g/min) compared to PCA and VBA (mean ± SD; 69.5 ± 18.2 and 58.3 ± 10.1 ml/100 g/min). CVR_rel_ was significantly higher in VBA and PCA (mean ± SD; 75.7 ± 26.8 and 59.0 ± 22.2) compared to MCA and ACA (mean ± SD; 47.9 ± 14.4 and 49.7 ± 17.3). In contrast, CVR_diff_ varied between 36.5 and 41.8 ml/100 g/min for all vascular regions, no significant differences between regions were found. Affected vascular (ACA and MCA) regions reached maximum CBF response to ACZ between 2.3 and 6.8 min later compared to unaffected regions (PCA and VBA). Differences were significant between ACA/MCA and VBA, (see Table 2). Median t_max_ (IQR) in minutes were 16.0 ± 10.9, 17.4 ± 10.4, 13.3 ± 5.6 and 11.6 ± 2.9 for ACA, MCA, PCA and VBA respectively. Static measurements significantly underestimated both CVR_rel_ and CVR_diff_ in all four regions. Mean CVR_rel_ underestimation ranged from 3.0 percentage points in PCA to 9.5 percentage points in VBA (see Table S4). Median CVR_diff_ underestimation ranged from 1.1 ml/100 g/min in PCA to 5.2 ml/100 g/min in VBA (see Table S3). Ambiguous fits were identified in four patients (one vascular region in each patient, see Fig. S4). Each fit was missing a local maximum.

**Table 1:** Descriptive statistics for model-derived parameters. Left and right vascular regions are pooled together and unaffected ACA/MCA regions were excluded, number of vascular regions included in each descriptive analysis is indicated by n.

Parameter	Mean (SD) or Median (IQR)			
	ACA (n = 32)	MCA (n = 34)	PCA (n = 36)	VBA (n = 38)
CBF_pre_ [ml/100 g/min]	79.5 ± 21.4	78.9 ± 18.8	69.5 ± 18.2	58.3 ± 10.1
CVR_rel_ [%]	49.7 ± 17.3	47.9 ± 14.4	59.0 ± 22.2	75.7 ± 26.8
CVR_diff_ [ml/100 g/min]^a^	38.5 ± 13.7	38.0 ± 13.4	36.5 ± 21.1	41.8 ± 26.4
t_max_ [min]^a^	16.0 ± 10.9	17.4 ± 10.4	13.3 ± 5.6	11.6 ± 2.9
RMSE [ml/100 g/min]^a^	3.8 ± 2.7	2.8 ± 2.7	2.8 ± 2.5	3.1 ± 3.2

Abbreviations: ACA, anterior cerebral artery; CVR, cerebrovascular reserve;.

IQR, interquartile range; MCA, middle cerebral artery; n, number of vascular regions; PCA, posterior cerebral artery; RMSE, root mean squared error; SD, standard deviation; VBA, vertebro-basilar artery.

a Data are expressed as median (IQR).

**Table 2:** Mean (SD) or median difference (IQR) between affected (ACA and MCA) and unaffected (PCA and VBA) for CBF_pre_, CVR_rel_, CVR_diff_ and t_max_. P-values were derived from a repeated measure ANOVA test with Sidak's correction or the Friedman test with Dunn's multiple comparisons test for a total of four comparisons. Comparisons with missing values were excluded, thus number of data points included were 30.

Model-based Parameter	Mean/Median (95% CI)	P-value
Mean/Median difference in model-derived parameters between ACA and PCA (n = 30)		

CBF_pre_ [ml/100 g/min]	10.5 (2.7 to 18.3)	0.006
CVR_rel_ [%]	−10.4 (−19.0 to −1.7)	0.01
CVR_diff_ [ml/100 g/min]^a^	−1.1 (−9.2 to 3.1)	0.99
t_max_ [min]^a^	2.3 (0.5 to 7.0)	0.77

Mean/Median difference in model-derived parameters between MCA and PCA (n = 30)		

CBF_pre_ [ml/100 g/min]	9.8 (2.5 to 17.1)	0.005
CVR_rel_ [%]	−11.8 (−17.9 to −5.7)	<0.001
CVR_diff_ [ml/100 g/min]^a^	−3.5 (−8.8 to 3.0)	0.92
t_max_ [min]^a^	3.9 (0.7 to 7.0)	0.11

Mean/Median difference in model-derived parameters between ACA and VBA (n = 30)		

CBF_pre_ [ml/100 g/min]	21.3 (14.7 to 27.9)	<0.001
CVR_rel_ [%]	−27.8 (−41.5 to −14.1)	<0.001
CVR_diff_ [ml/100 g/min]^a^	−7.3 (−13.4 to 4.7)	0.99
t_max_ [min]^a^	4.2 (1.4 to 10.9)	0.001

Mean/Median difference in model-derived parameters between MCA and VBA (n = 30)		

CBF_pre_ [ml/100 g/min]	20.6 (14.6 to 26.7)	<0.001
CVR_rel_ [%]	−29.2 (−41.7 to −16.8)	<0.001
CVR_diff_ [ml/100 g/min]^a^	−6.9 (−13.8 to 0.8)	0.14
t_max_ [min]^a^	6.8 (3.6 to 9.6)	<0.001

Abbreviations: ACA, anterior cerebral artery; CVR, cerebrovascular reserve;.

IQR, interquartile range; MCA, middle cerebral artery; n, number of vascular regions; PCA, posterior cerebral artery; SD, standard deviation; VBA, vertebro-basilar artery.

a Data are expressed as median (95% CI). P-values derived using Friedman's test.

**Fig. 2:**
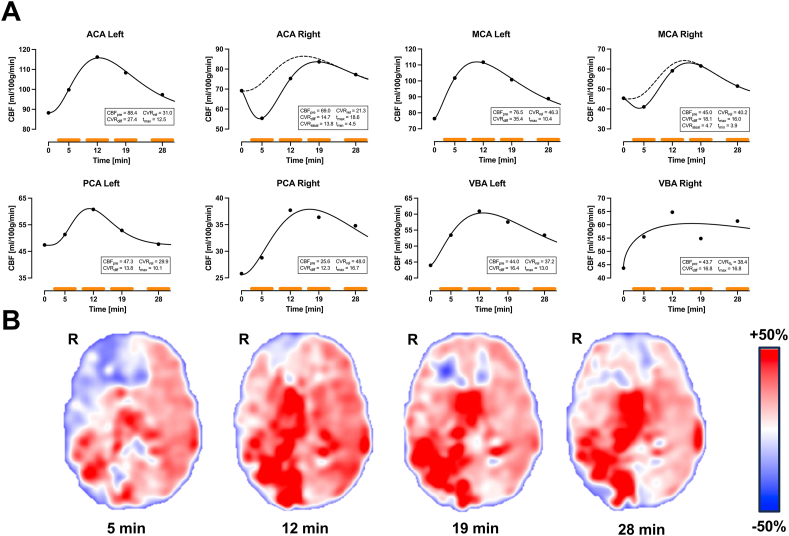
Male patient (45 years old) with bilateral MMD (Suzuki III). **(A)** Reversible intracerebral steal was present in the right ACA and right MCA region whereas a non-steal response was seen in the remaining vascular regions. CVR_steal_ ranged between 4.0 and 13.8 ml/100 g/min and t_min_ between 4.9 and 5.5 min. Dashed line indicates the non-steal model using the corresponding parameters from the reversible steal model fit. **(B)** Parametric CVR maps demonstrates reversible intracerebral steal in the right frontal region. Orange bars indicate the acquisition time for each CBF measurement. Abbreviations: ACA, anterior cerebral artery; CBF, cerebral blood flow; MCA, middle cerebral artery; PCA, posterior cerebral artery; R, right; VBA, vertebra-basilar artery.

**Fig. 3:**
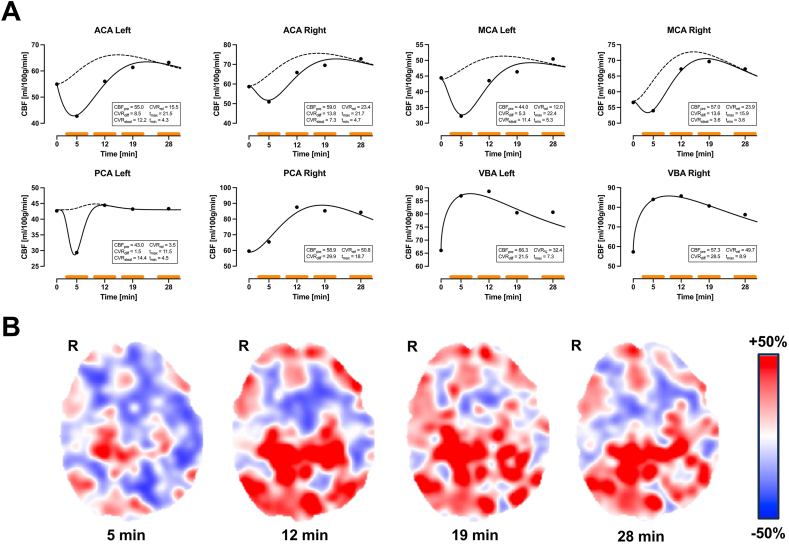
Female patient (54 years old) with bilateral MMD (Suzuki IV). Revascularization surgery (multiple burr holes) performed on both hemisphere at difference occasions. Reversible intracerebral steal was present in left and right ACA and MCA and also in left PCA. Normal response was present in remaining vascular regions. CVR_steal_ ranged between 3.6 and 14.6 ml/100 g/min and t_min_ between 3.6 and 5.3 min. Dashed line indicates the non-steal model using the corresponding parameters from the reversible steal model fit. **(B)** Reversible intracerebral steal is demonstrated in cortical regions bilaterally. Orange bars indicate the acquisition time for each CBF measurement. Abbreviations: ACA, anterior cerebral artery; CBF, cerebral blood flow; MCA, middle cerebral artery; PCA, posterior cerebral artery; R, right; VBA, vertebra-basilar artery.

### 3.3 Exploratory analysis of reversible intracerebral steal

We noted seven vascular regions with reversible intracerebral steal in two patients which encouraged for non-statistical exploratory analysis using an extended version of the model where the results are presented and discussed on a case-by-case basis.

#### 3.3.1 Case 1

45-year-old male with unilateral MMD (Suzuki III bilaterally). Reversible intracerebral steal was present in the right ACA and right MCA region whereas a non-steal response was seen in the remaining vascular regions. CVR_steal_ ranged between 4.7 and 13.8 ml/100 g/min and t_min_ between 3.9 and 4.5 min. Graphs and CVR maps are presented in Fig. 2.

#### 3.3.2 Case 2

54-year-old female with bilateral MMD occlusions in ACA and MCA, and stenosis/occlusion in PCA (Suzuki IV bilaterally). Revascularization surgery (multiple burr holes) performed on both hemisphere at difference occasions before the examination. Reversible intracerebral steal was present in left and right ACA and MCA and also in left PCA. Normal response was present in remaining vascular regions. CVR_steal_ ranged between 3.6 and 14.4 ml/100 g/min and t_min_ between 3.6 and 5.3 min. Graphs and CVR maps are presented in Fig. 3.

## 4 Discussion

The gamma-variate function was chosen because it captures the key features of the observed CBF response to ACZ: a rise from baseline, a single peak, and a gradual decay as the pharmacological effect wears off and CBF returns to CBF_pre_. In this respect, the gamma-variate is a more plausible descriptive model than the bi-exponential used in previous work (Fahlstrom et al., 2024), which had an upper limitation on possible CVR estimates. Although this limitation of the bi-exponential could in principle be resolved by adding another free parameter, doing so would have equalized the degrees of freedom between the two models. For that reason, and given the lack of physiological interpretation of the bi-exponential parameters, we chose not to include a direct comparison between the two models. In its original formulation, the gamma-variate function has shape (*α*) and scale (*β*) parameters that are coupled, making it difficult to anticipate how the function changes as these parameters are altered (Madsen, 1992). This issue is eliminated in the Madsen parametrization, where the fitted parameters directly correspond to physiologically meaningful features of the response: the peak CBF increase (CVR_diff_) and the time at which this peak occurs (t_max_). Initial values and constraints for fitting can therefore be estimated from physiologically reasonable ranges reported in the literature.

The temporal response to ACZ is well documented in current literature (Fahlstrom et al., 2024). However, statistical deviations of the CBF values (which are sampled from a distribution of CBF values within specific regions) or artefacts such as motion or ineffective labeling may introduce variations which are not secondary to the vasodilatory effect of ACZ which may affect one or several CBF values. The curve fitting performance is especially sensitive to a missing local maximum which is a common reason for ambiguous fits. Four cases are presented in Fig. S4 with the last CBF value at 28 min post-ACZ injection being the highest value. The line from the curve fit does not reach a maximum within the measurement period (0 to 31 min) and the best-fit t_max_ values (see text box in Fig. S4) are exceptionally high compared to average values (see Table 1). CVR_diff_ seems less affected, but in general curve fits need to be reviewed visually. The issue could be addressed by adding a CBF measurement after 28 min. However, ACZ is diuretic and to include further time points after 28 min would increase the probability for interruptions of the scanning. Interestingly, similar responses have been reported in patients with steno-occlusive disease before, based on ^15^O-water PET acquired at 5, 15 and 30 min post-ACZ injection (Kobayashi et al., 2021). These patients were classified with abnormally decreased cerebral metabolic rate of oxygen based on a^15^O_2_-PET acquisition before ACZ injection. This may therefore describe a temporal CBF response specific to some patients, which may hold clinical value.

### 4.1 Model-based parameters

The necessity of applying a mathematical model to several acquired CBF measurements after ACZ injection strongly relies on the hypothesis that the temporal response of CBF to ACZ differs between healthy controls and patients with cerebrovascular diseases, as well as between affected and unaffected vascular regions within patients. However, it is important to mention that this hypothesis is only supported in a handful of studies (Dogra et al., 2023; Fahlstrom et al., 2024; Ficzere et al., 1997; Hartkamp et al., 2012; Stoll et al., 1996). This small number of publications can partly be explained by lack of dynamic CVR imaging methods, i.e. static CVR measures are more available in the current literature. Furthermore, it is important to note that there is currently no consensus concerning this hypothesis or how it may affect CVR measurements in a clinical setting.

With that in mind, we found t_max_ values (see Table 1) at 16.0 and 17.4 min in affected- (ACA and MCA, respectively) and 13.3 and 11.6 min in unaffected (PCA and VBA, respectively) regions. Mean differences reach significance for comparisons to VBA (see Table 2), this suggests longer t_max_ in affected-compared to unaffected regions, further adding support to the above-mentioned hypothesis. The model therefore enables estimation of CVR independently of variations in t_max_. The model-based CVR (CVR_diff_) is defined as the subtraction between the maximum CBF after ACZ injection compared to pre-injection values. The common definition of CVR is the ratio in percentage (CVR_rel_) relative to CBF_pre_ (Vagal et al., 2009) which is easily calculated using the derived best-fit parameter values. We found CVR_rel_ in affected vascular regions around 47 to 50%, which is slightly higher compared to others and 59.0% to 75.7% in unaffected vascular regions (Fahlstrom et al., 2021). One potential explanation could be that CVR_rel_ is underestimated using a single CBF measurement most commonly at 15 min after ACZ injection (Fahlstrom et al., 2021).

We found values of CVR_diff_ ranging from 36.5 to 41.8 ml/100 g/min across all vascular regions. These values are comparably higher compared to reported by others. Puig et al. (2020) reported a mean CVR_diff_ in GM of 21.9 ± 8.1 ml/100 g/min (mean ± sd) in healthy volunteers using ASL. Post-ACZ measurement was performed 20 min after injection. Yamauchi et al. (2002) reported mean CVR_diff_ in patients with steno-occlusive disease and healthy volunteers using ^15^O-water PET, 10 min after ACZ injection. Mean CVR_diff_ was lower in ipsilateral hemisphere (mean ± sd, 12.6 ± 7.9 ml/100 g/min) compared to contralateral (mean ± sd, 16.8 ± 7.0 ml/100 g/min). Mean CVR_diff_ in healthy volunteers was 14.7 ± 3.6 ml/100 g/min (mean ± sd). CVR_diff_ is rarely reported in literature, hence comparisons remain difficult given different imaging- and image analysis methods.

### 4.2 Reversible intracerebral steal

Intracerebral steal secondary to vasodilatory challenge has been described before in diverse patient groups using different imaging modalities and different vasodilatory substances (Hartkamp et al., 2019; Kobayashi et al., 2021; Kuwabara et al., 1995; Nariai et al., 1998; Poublanc et al., 2013; Sobczyk et al., 2014; van Grinsven et al., 2023; Vorstrup et al., 1986). The general definition of an intracerebral steal is reduced CBF (negative CVR) in an affected vascular region with increased CBF (positive CVR) in unaffected vascular regions, i.e. blood flow is thought to be rerouted from a region of exhausted CVR to a region of relatively preserved CVR (Arteaga et al., 2014; Nariai et al., 1998; Poublanc et al., 2013; van Grinsven et al., 2023; van Niftrik et al., 2023), which implies that no further vasodilation is possible in the exhausted region.

The reversible steal model is adapted from the two-component gamma-variate formulation of Davenport (1983), originally derived for tracer recirculation. We simplify it to the present application by setting the time delay between the two components to zero and giving them opposite signs, producing the dip-then-recovery pattern characteristic of reversible intracerebral steal. In this study we present two cases with reversible intracerebral steal present in seven vascular regions (see Fig. 2, Fig. 3). These cases point toward two significant implications: intracerebral steal does not necessarily equate to an exhausted CVR capacity; timing of the CBF/CVR measurements after ACZ injection is of high importance. The former has also been suggested by van Niftrik et al. (van Niftrik et al., 2023) by comparing CVR derived from BOLD to motor-tasked BOLD-functional MRI in patients with steno-occlusive disease using CO_2_ as vasodilatory stimulus. Both Kuwabara et al. (1995) and Kobayashi et al. (2021) observed reversible intracerebral steal using ^15^O-water PET together with ACZ in patients with steno-occlusive disease. Although they did not discuss possible implications in detail, the results presented provide evidence of the presence of reversible intracerebral steal. Of note, reversible intracerebral steal in Kobayashi et al. (2021) was reported in a subgroup of patients with abnormally high cerebral blood volume measured with ^15^O-gas PET. In further support, Poublanc et al. (2013) also reported reversible intracerebral steal using BOLD and CO_2_ in patients with steno-occlusive disease. It should be noted that reversible intracerebral steal is reported on a case basis within groups of patients using different imaging modalities and vasodilatory substances; hence, it is difficult to draw any specific conclusions on what underlying hemodynamic and structural circumstances set up the conditions for reversible steal. However, given that competition for limited blood supply is thought to be a driver of the steal phenomenon, its reversal may imply the presence of additional collateral pathways (Sobczyk et al., 2014). To confirm this hypothesis, further exploration of reversible steal and steal phenomena in general is necessary, but higher temporal resolution of dynamic measurements is warranted.

### 4.3 Implementation of future studies

Our observations highlight an important methodological caveat in ACZ-augmented hemodynamic studies where typically only one CBF/CVR measurement is performed post-injection, often at ∼15 min (Fahlstrom et al., 2021). Our t_max_ results quantify this concern: affected regions reached a peak CBF response 2 to 7 min later than unaffected regions. A direct comparison in our data showed that static measurements at 16 min post-ACZ systematically underestimated model-based CVR in all four vascular regions, illustrating that single-time-point acquisitions is sensitive to regional variations in the temporal response regardless of whether the region is affected or unaffected. Reversible intracerebral steal places additional demands on acquisition timing. In the two cases presented here, t_min_ occurred within the first 5 min post-injection, while recovery above CBF_pre_ was observed between 12 and 15 min. Given the temporal dynamics, this phenomenon might be missed with single measurement protocols (Kobayashi et al., 2021; Kuwabara et al., 1995) and its detection requires sampling at both early and later time points. Extension to arterial transit time (ATT), which is available from MD-ASL and is known to decrease following ACZ (Zhao et al., 2021, 2022), is a natural direction for future work.

### 4.4 Limitations

Statistical variations in individual CBF measurements may produce erroneous curve fits which can affect the values of the best-fit parameters (this is discussed in more detail above). Most included patients were female, and the cohort comprised both patients who had undergone prior revascularization surgery and those who had not at the time of examination. Thus, the patient group included in this study represents a heterogeneous population, and any comparisons to other study populations should be done with caution. The cohort size was insufficient to support meaningfully powered subgroup analyses by age, sex or surgical history. Three patients presented with MMS rather than MMD, all with unilateral disease. Since contralateral ACA/MCA regions were excluded, the analysis was restricted to affected ACA/MCA regions where hemodynamic compromise distal to the stenosis should be comparable between MMD and MMS (Juttukonda and Donahue, 2019). We only identified patients presenting with reversible intracerebral steal, hence other steal responses were not included in the modelling framework.

The reversible steal model introduces an additional unknown parameter matching the available number of data points. To enable curve fitting, CBF_pre_ was set to the measured pre-ACZ injection CBF values. Moreover, the values of CVR_steal_, t_min_, CVR_diff_ and t_max_ were derived by numeric approximation hence the physiological interpretation is not straight forward as compared to the non-steal model. These are clear limitations of the reversible steal model which was only applied on a few cases; hence conclusions should be interpreted with caution.

MD-ASL data is acquired during a period of approximately 6 min including a calibration image. As such, the resulting CBF measurement can be considered a weighted average taking into account the effects of ACZ on CBF, moreover the weighting is further complicated by the multiple time points acquisition.

Arterial transit time (ATT) is known to be reduced secondary to ACZ injection (Zhao et al., 2021, 2022) and is typically prolonged in patients with steno-occlusive diseases such as MMD (Mikayama et al., 2025; Togao et al., 2023; Zhao et al., 2022), both these factors can confound the CBF measurement. In this study, an advanced MD-ASL sequence was paired with a recently developed quantification algorithm (SSVB) that offers increased accuracy and robustness over existing methods (Kirk et al., 2025; Togao et al., 2023), particularly for subjects with elongated ATT or measurements during changing ATT conditions.

## 5 Conclusion

We present a parameterized gamma-variate framework for modelling the temporal CBF response to ACZ, with fitted parameters that admit physiological interpretation.

Because t_max_ is a fitted parameter, CVR estimation is robust to variations in t_max_ across regions and patients. The framework may be applied to other imaging modalities and vasodilatory stimuli. Furthermore, affected vascular regions have a delayed temporal response of CBF compared to unaffected vascular regions. This supports the use of dynamic CBF measurements to overcome confounding effects of t_max_ variations. The model was extended to include reversible intracerebral steal cases, which may provide groundwork for further exploration of this phenomenon. Future work should include healthy controls and other patient cohorts, and address potential clinical applications.

## Declaration of generative AI and AI-assisted technologies in the writing process

During the preparation of this work, the authors used Claude to improve readability and language. After using this tool, the authors reviewed and edited the content as needed and take full responsibility for the content of the publication.

## Funding

This work was supported by the Erik, Karin and Gösta Selanders Foundation, the Swedish Stroke Association and the Swedish Brain Foundation. The supporters had no role in conceptualization, design, data collection analysis, decision to publish, or preparation of the manuscript.

## CRediT authorship contribution statement

**Joao M. Sousa:** Formal analysis, Investigation, Methodology, Software, Writing – review & editing. **Teodor Svedung Wettervik:** Conceptualization, Investigation, Methodology, Writing – review & editing. **Fartein Velle:** Conceptualization, Investigation, Writing – review & editing. **Alex A. Bhogal:** Investigation, Methodology, Writing – review & editing. **Johan Berglund:** Conceptualization, Investigation, Methodology, Resources, Software, Writing – review & editing. **Per Enblad:** Conceptualization, Supervision, Writing – review & editing. **Johan Wikström:** Conceptualization, Supervision, Writing – review & editing. **Anders Lewén:** Conceptualization, Funding acquisition, Supervision, Writing – review & editing. **Markus Fahlström:** Conceptualization, Data curation, Formal analysis, Funding acquisition, Software, Supervision, Validation, Visualization, Writing – original draft.

## Declaration of competing interest

The authors declare that they have no known competing financial interests or personal relationships that could have appeared to influence the work reported in this paper.

## Data Availability

Anonymized data from this study is available, upon reasonable request, from the corresponding author if it does not violate GDPR and local ethics committee rules.
