# Supplementary material for: A parameterized gamma-variate function can describe the temporal response of cerebral blood flow after Acetazolamide injection in Moyamoya patients
Source: Neuroimage Rep. 2026 May 21;6(2):100359. doi: 10.1016/j.ynirp.2026.100359 (PMC13218246; doi:10.1016/j.ynirp.2026.100359)
Supplement: Multimedia component 1 [file mmc1.docx]

Supplemental material

**Table S1:** Acquisition parameters

| **Parameter** | **Value** | **Unit** |
| --- | --- | --- |
| **MD-ASL** | | |
| Label Duration | 400, 900, 2000, 2000, 2000, 2000 | ms |
| Post Label Duration | 100, 100, 500, 1500, 2500, 3000 | ms |
| Background suppression | 4 pulses, optimized scheme |  |
| Excitation/refocusing FA | 90/180 | degrees |
| Acquired voxel size | 3.75 x 3.75 x 6 | mm |
| Reconstructed voxel size | 3.2 x 3.2 x 6 | mm |
| Field-of-view | 256 x 243 x 132 | mm^3^ |
| SENSE factor | 1.5 (anterior-posterior direction) |  |
| Slices | 22 |  |
| Turbo Spin Echo factor | 12 |  |
| EPI factor | 13 |  |
| TE | 15 | ms |
| TR | Variable | ms |
| Half scan | 1 x 0.8 (right-left, feet-head direction) |  |
| iMSDE | TE 12.5  Venc 5 cm/s in all 3 directions |  |
| Scan time | MD-ASL 5:15 min  Calibration Image 00:54 (TR 3000 ms) |  |

Abbreviations: EPI, echo planar imaging; FA, flip angle; iMSDE, improved motion-sensitized driven-equilibrium; MD-ASL, multi-delay arterial spin labelling; TE, echo time; TR, repetition time

**Table S2:** Patient demographics

| **Subject** | **Age** | **Sex** | **Diagnosis** | **Affected Hemisphere (L/R)** | **Previous surgery** | **Comment** |
| --- | --- | --- | --- | --- | --- | --- |
| 01 | 32 | Female | MMD | V / I | MBH R |  |
| 02 | 42 | Female | MMS | - / II |  |  |
| 03 | 33 | Female | MMD | III / IV | MBH L/R |  |
| 04 | 29 | Female | MMD | IV / IV | Bypass / MBH R | Ambiguous Left ACA |
| 05 | 57 | Female | MMD | III / III |  |  |
| 06 | 54 | Female | MMS | - / III |  |  |
| 07 | 54 | Female | MMD | IV / IV | MBH L/R | rICS ACA L/R, MCA L/R, PCA L |
| 08 | 30 | Male | MMS | - / III |  |  |
| 09 | 50 | Female | MMD | V / V |  |  |
| 10 | 39 | Female | MMD | IV / IV |  | Ambiguous Left PCA |
| 11 | 44 | Female | MMD | III / III | MBH L |  |
| 12 | 24 | Female | MMD | IV / III | Bypass L |  |
| 13 | 51 | Female | MMD | III / III |  | Excluded |
| 14 | 32 | Male | MMD | III / III | MBH L/R |  |
| 15 | 71 | Female | MMD | III / III | By-pass L |  |
| 16 | 48 | Female | MMD | III / III | MBH L |  |
| 17 | 30 | Male | MMD | III / III | MBH L |  |
| 18 | 54 | Female | MMD | - / III |  |  |
| 19 | 32 | Female | MMD | III / IV | By-pass L/R | Ambiguous Left ACA |
| 20 | 45 | Male | MMD | III / III |  | rICS Right ACA Right MCA |
| 21 | 43 | Male | MMD | III / III | MBH R |  |
| 22 | 11 | Male | MMD | III / II | MBH L/R | Excluded |
| 23 | 63 | Male | MMD | III / IV |  | Excluded |
| 24 | 10 | Female | MMD | II / II | MBH L/R | Ambiguous Right PCA |

Abbreviations: rICS, reversible intracerebral steal; F, female; L, left; M, male; MBH, multiple burr hole surgery; MMD, Moyamoya Disease; MMS, Moyamoya syndrome; R, right

**Table S3.** Comparison of static (CVR_diff, 16min_) and model-based CVR_diff_ per vascular region. Values expressed as median (IQR); differences reported as median difference with 95% CI. P-values from Wilcoxon signed-rank test.

| **Region** | **n** | **CVR_diff, 16min_** | **CVR_diff_** | **Difference^a^** | **P-value** |
| --- | --- | --- | --- | --- | --- |
| ACA | 32 | 33.4 ± 14.8 | 38.5 ± 13.7 | 4.0 (2.2 to 6.5) | <0.001 |
| MCA | 34 | 33.7 ± 17.2 | 38.0 ± 13.4 | 3.3 (2.7 to 4.9) | <0.001 |
| PCA | 36 | 30.9 ± 22.5 | 36.5 ± 21.1 | 1.1 (-0.5 to 3.7) | <0.001 |
| VBA | 38 | 34.9 ± 25.0 | 41.8 ± 26.4 | 5.2 (2.9 to 7.8) | <0.001 |

Abbreviations: ACA, anterior cerebral artery; CVR, cerebrovascular reserve; IQR, interquartile range; MCA, middle cerebral artery; n, number of vascular regions; PCA, posterior cerebral artery; VBA, vertebrobasilar artery

^a^Median differences calculated as CVR_diff_ minus CVR_diff, 16min_. Positive values indicate underestimation by the static measurement.

**Table S4.** Comparison of static (CVR_rel, 16min_) and model-based CVR_rel_ per vascular region. Values expressed as mean (SD); differences reported as mean with 95% CI. P-values from paired t-test.

| **Region** | **n** | **CVR_rel, 16min_** | **CVR_rel_** | **Difference^a^** | **P-value** |
| --- | --- | --- | --- | --- | --- |
| ACA | 32 | 43.9 ± 15.9 | 47.9 ± 17.3 | 5.8 (3.8 to 7.9) | <0.001 |
| MCA | 34 | 43.4 ± 13.6 | 47.9 ± 14.4 | 4.6 (2.9 to 6.3) | <0.001 |
| PCA | 36 | 56.0 ± 22.4 | 59.0 ± 22.2 | 3.0 (1.0 to 5.2) | <0.001 |
| VBA | 38 | 66.2 ± 28.0 | 75.7 ± 26.8 | 9.5 (6.7 to 12.2) | <0.001 |

Abbreviations: ACA, anterior cerebral artery; CVR, cerebrovascular reserve; MCA, middle cerebral artery; n, number of vascular regions; PCA, posterior cerebral artery; SD, standard deviation; VBA, vertebrobasilar artery

^a^Mean differences calculated as CVR_rel_ minus CVR_rel, 16min_. Positive values indicate underestimation by the static measurement.

**Figure S1:** The maximum CBF response after ACZ injection (CBF_max_) subtracted by CBF before ACZ injection (CBF_pre_) at time t_max_ yields CVR_diff_ in ml/100g/min (A). CBF_pre_ subtracted by the minimum CBF response following ACZ injection (CBF_min_) at time t_min_ yields CVR_steal_ in ml/100g/min (B). The figure presents the so-called dip-then-recovery pattern.

Abbreviations: ACZ, acetazolamide; CBF, cerebral blood flow; CVR, cerebrovascular reserve

**Figure S2:** Vascular territory regions for a representative patient.

Abbreviations: ACA, anterior cerebral artery; MCA, middle cerebral artery; PCA, posterior cerebral artery; VBA, vertebrobasilar artery

**Figure S3:** Flowchart describing patient inclusions and exclusion and the number of vascular regions included in the statistical analysis distributed over type of vascular region.

Abbreviations: ACA, anterior cerebral artery; MCA, middle cerebral artery; PCA, posterior cerebral artery; VBA, vertebrobasilar artery

**Figure S4:** Presents identified ambiguous fits based on outlier analysis of t_max_. All fits are missing a local maximum which will make it difficult for the model to estimate reasonable t_max_ values.

Abbreviations: CBF, cerebral blood flow; CVR, cerebrovascular reserve
